# The glue that holds us together

**DOI:** 10.1038/s41467-023-44476-5

**Published:** 2024-01-04

## Abstract

As part of our tenth-anniversary celebrations, the editorial team at *Nature Communications* wanted to hear from early career researchers who have published with us. We asked the early career researchers to tell us in an essay what is amazing about the research question(s) that drove them and the highs—and lows—of the journey from hypothesis to publication.

**Figure:**
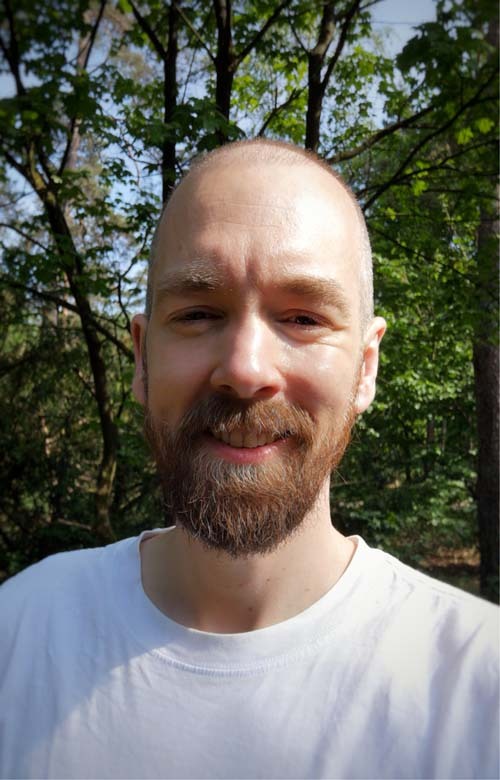
Tim Koopmans

Tim Koopmans obtained his PhD in the lab of Prof. Gosens at the University of Groningen, Netherlands, where he studied the re-emergence of developmental pathways as drivers of airway fibrosis. With his gained experience, he continued his career in tissue fibrosis at the Helmholtz Institute in Munich, Germany. As a post-doc in the lab of Dr. Rinkevich, he studied abdominal fibrosis and the abnormal fusion of organ surfaces that develop post-operation. Currently, he is working at the Hubrecht Institute in the Netherlands, where he is focused on the scarring response in the ischemic heart.

Q: As an early career researcher and an author of a paper published by *Nature Communications*^1^, tell us about the journey you’ve been on?

A: As a native Dutch I moved to the capital of Bavaria in Germany in January 2017, where I joined the lab of Dr. Rinkevich to work as a fresh post-doc. My PI’s work on tissue regeneration and scarring held a great appeal to me, in particular his work on the surface mesothelium. To me, the mesothelium was somewhat of an enigma, a structure that I hadn’t seemed to have picked up on during my college years. For those wondering, the mesothelium is essentially an epithelial monolayer that lines the surfaces of our cavities, as well as the organs within them. The constituent cells secrete fluid which accommodates organ movement and decreases friction. More interesting perhaps is the intrinsic predisposition of mesothelia to adopt mesenchymal properties and contribute to tissue fibrosis upon exposure to a stressor. This predisposition is especially problematic during surgeries, when organs are being manipulated by the surgeon and tissues are dehydrated by the circulating air or exposed to foreign materials. The resulting breach in mesothelial integrity can result in what is called surgical adhesions: thick fibrous bands that glue organs together, causing pain, organ dysfunction, and, in rare cases, death. Surgical adhesions are prevalent and develop in more than 90% of abdominal operations, at times requiring follow-up surgery to correct adhesion-related complications. This potentially cyclic nature of repeated trauma adds an enormous burden to the healthcare system, with an estimated cost of over $1 billion a year in the United States alone. Yet, surgical adhesions have received very little attention from the scientific community.

In the world of pathologies, adhesions differ from most other conditions in that they are almost always caused by human handling, making them predictable in their onset. Whereas many conditions need to be treated when they have already manifested, mesothelial adhesions can be potentially ‘treated’ even before they develop. Within that framework, any potential therapeutic is best delivered immediately after the injury trigger, and as such, the efforts of my research group were directed towards the early mechanisms that initiate the adhesion cascade. Although we were planning to use single-cell RNA sequencing to investigate potential genes that drive adhesions, the logistics of the experiment required us to wait several months before we could obtain any data. To put our waiting time to good use, we undertook several small-scale screening studies in which we tried to block different cell adhesion molecules. Prior to this, we had developed an in vivo as well as in vitro adhesion model that would be perfect to use for this purpose. We figured cell adhesion molecules would be the logical choice when searching for the mechanistic root (how else do cells bind to one another?), and so it would just be a matter of finding the right combination. However, none of our attempts proved anywhere near successful, and adhesions developed as readily as they did under control conditions. Intrigued and frustrated at the same time, we ventured toward other possible protein families that we could think of, but we always hit a dead end. Until one day we found our golden nugget. I remember using the confocal microscope to look at some peritoneal tissue coming from a mouse that had received adhesion surgery. This was not the first time we were capturing whole-mount images to visualize the mesothelium in three dimensions, but the first time we looked at an early time point, 16 hours after the surgery to be exact. When I first peeked through the ocular, I couldn’t believe my eyes and initially figured that I was looking at the wrong side of the tissue. Mesothelial cells are normally round, sitting in a cobblestone arrangement like most other epithelia and adopting a fibroblast-like morphology when injured. Yet, these cells didn’t look round at all, nor did they look like fibroblasts. If anything, they looked more like astrocytes, expressing a host of protrusions in a radial pattern. By chance, in the same month, a co-worker had observed the exact same morphology in our in vitro culture using pleural mesothelial, suggesting this may be a conserved mechanism of mesothelial cells. This simple observation completely changed the way we were thinking and prompted us to start asking a different question: could it be that injured mesothelia adhere to other cells simply by physically grabbing onto them? With that in mind, we waited for the RNA sequencing data to come in, and, sure enough, we were able to confirm our observations on a transcriptional level. The data indicated that there is a short window of approximately 8–16 hours after the injury during which mesothelial cells upregulate a large array of cytoskeletal proteins that allow them to move and tether to opposing cells via long dynamic protrusions. We found that inhibiting key proteins within this cytoskeletal family could completely block the development of adhesions in both in vitro and in vivo models.

This observation became the cornerstone of our paper^1^. We were the first to show that adhesions can be fully prevented by focusing on the early mechanisms within the mesothelium. Up to that point efforts had only been partially successful, in part because the emphasis had always been on the fibrosis aspect of adhesions, which occurs secondary to the adhesion itself. Accordingly, fibroblasts or mesothelial-derived fibroblasts have been the main focus of the study. Our findings suggest that the therapeutic window of opportunity to prevent surgical adhesions in the clinic may be limited to only a few hours after the original stressor has been applied. Once mesothelial cells commit to mesenchymal differentiation and adopt fibroblast-like properties, it is likely either too late or more complicated to achieve successful preventative outcomes.
